# Abstract of Meteorological Observations for December, 1855, Made at Philadelphia, Pa.

**Published:** 1856-02

**Authors:** James A. Kirkpatrick


*Abstract of Meteorological Observations for December*, 1855, *made at Philadelphia, Pa. Latitude* 39° 57′ 28″ *N., Longitude* 75° 40″ 10′ *W. from Greenwich.* By PROF. JAMES A. KIRKPATRICK.

| 1855. December. | BAROMETER. Daily Mean | BAROMETER. Mean Daily Range. | THERMOM. Daily Mean | THERMOM. Mean Daily Range | Dew Point 2 P.M. | Force of Vapor 2 P.M. | Rel. Humid. 2 P.M. | Rain. | Prevailing Winds. | Remarks. |
|---|---|---|---|---|---|---|---|---|---|---|
| - | Inches. | Inches | Deg. | Deg. | Deg. | Inches. | Hunds. | Inch. | Points. | |
| 1 | 29.821 | .193 | 41.3 | 4.7 | 35.8 | .211 | .63 | | SW. | Clear. |
| 2 | 29.493 | .332 | 42.5 | 3.5 | 42.2 | .269 | .89 | | SW. | M. and aft. fog; ev. clear. |
| 3 | 29.728 | .239 | 40.8 | 5.0 | 24.0 | .129 | .41 | | W | Clear. |
| 4 | 29.982 | .254 | 41.5 | 3.7 | 29.8 | .165 | .49 | | SW. | M. clear; aft. & ev. cloudy. |
| 5 | 30.004 | .030 | 41.8 | 2.3 | 29.5 | .162 | .45 | | WSW. | M. fog; aft. & ev. clear. |
| 6 | 29.958 | .046 | 42.0 | 1.2 | 29.2 | .161 | .42 | | WSW. | Clear. |
| 7 | 30.088 | .130 | 41.2 | 4.2 | 24.7 | .133 | .42 | | NW. | Clear. [6 P. M. *hail.* |
| 8 | 30.039 | .130 | 39.7 | 4.5 | 26.2 | .142 | .51 | | W. | M. clear; aft. & ev. cloudy. |
| 9 | 29.175 | .864 | 52.0 | 12.3 | 54.2 | .421 | .91 | 1.456 | (Var.) | Cl'dy; m. & ev. *rain. Bar.* |
| 10 | 29.391 | .376 | 37.3 | 14.7 | 20.8 | .112 | .44 | | WSW. | Clear. [*lowest* 28.946. |
| 11 | 29.908 | .517 | 31.3 | 6.0 | 17.2 | .095 | .49 | | WNW. | Cloudy. [*highest* 30.445. |
| 12 | 30.378 | .470 | 30.7 | 2.3 | 26.7 | .145 | .74 | | WNW. | M. & aft. cl'r; ev. cl'y. *Bar.* |
| 13 | 30.219 | .159 | 34.3 | 3.7 | 42.7 | .186 | .82 | 0.163 | NE. | Cl'y; m. *snow*; ev. drizzling |
| 14 | 3 .303 | .084 | 32.2 | 2.2 | 31.5 | .177 | .86 | | NE. | Cloudy; ev. *rain.* [*sleet.* |
| 15 | 30.013 | .290 | 44.8 | 12.7 | 45.5 | .305 | .92 | | NE. | Cl'dy; drizzling all day. |
| 16 | 29.742 | .271 | 53.0 | 8.8 | 57.3 | .471 | .91 | } 1.411 | SW. | Cl'y; m. *rain*, ev. drizzling. *Therm. highest* 60° |
| 17 | 29.766 | .083 | 48.0 | 6.0 | 35.6 | .209 | .53 | | WNW. | M. clear, aft. and ev. cl'dy. |
| 18 | 30.006 | .239 | 37.3 | 10.7 | 19.4 | .105 | .40 | | W. | Clear. |
| 19 | 30.056 | .114 | 33.2 | 4.5 | 25.9 | .140 | .62 | | NE. | Cloudy. |
| 20 | 30.131 | .075 | 32.7 | 1.2 | 24.0 | .129 | .60 | | NW. | M. & aft. cloudy. ev. clear. |
| 21 | 30.017 | .114 | 38.0 | 5.3 | 30.9 | .173 | .60 | | (Var.) | Cloudy. |
| 22 | 29.687 | .330 | 47.5 | 9.5 | 45.0 | .299 | .92 | 0.636 | (Var.) | *Raining* all day. |
| 23 | 29.716 | .156 | 52.8 | 7.3 | 45.9 | .310 | .64 | | SW. | M & ev. cloudy, aft clear. |
| 24 | 30.027 | .311 | 41.8 | 11.0 | 27.7 | .151 | .57 | 0.006 | NW. | Cl'y, m. *rain*, 11¼ A. M. *hail.* Tem. 42° dry bulb, & wet |
| 25 | 29.751 | .276 | 37.8 | 4.7 | 35.9 | .211 | .90 | 0.916 | NNE. | *Raining* all day. [bulb 32° |
| 26 | 29.922 | .408 | 27.2 | 10.7 | 15.6 | .088 | .55 | | W. | M. cl'dy, aft. & ev. clear. 9 A. M. a few flakes of snow. |
| 27 | 30.413 | .491 | 20.0 | 8.2 | 17.2 | .095 | .74 | | W. | [illegible]. & aft. cl'y, ev. cl'r. *Ther. lowest* 13.° |
| 28 | 30.053 | .360 | 31.7 | 11.7 | 28.0 | .153 | .79 | | (Var.) | Cl'dy. m. *snow.* |
| 29 | 29.901 | .152 | 21.2 | 10.2 | 19.8 | .107 | .92 | 0.418 | NE. | [illegible]l'y. 10 A. M. to 2 P. M. *snow;* 4 P. M. and night *hail* and |
| 30 | 29.741 | .298 | 25.0 | 3.8 | 18.3 | .100 | .67 | | W. | Clear. [*snow.* |
| 31 | 39.051 | .310 | 21.8 | 3.2 | 15.1 | .086 | .64 | | WNW. | Cloudy. |
| Means for Dec., 1855 | 29.919 | .261 | 37.5 | 6.4 | 30.0 | .182 | .66 | 5.006 | N. 76° 54′ W. 47-100. | |
| Means for Dec., 5 yrs. | 29.934 | .217 | 34.5 | 6.4 | 30.6 | | .70 | 3.884 | N. 54° 12′ W. 49-100. | |
| Ann'l Means 1855 | 29.872 | .155 | 54.5 | 5.4 | 44.2 | | 60. | 44.653 | N. 63° 26′ W. 39-100. | |
| Ann'l Means 4 yrs. | 29.908 | | 54.2 | | 45.9 | | | 44.761 | N. 66° 16′ W. 37-100. | |

